# Summer Drinking

**Published:** 1873-02

**Authors:** 


### SUMMER DRINKING.

Drinking a great deal of cold water in warm weather disturbs the whole system; it gives a filled up feeling; it promotes large perspirations; this debilitates, and increases the liability to colds. The earlier in the day a person drinks, the greater will be the thirst. A whole glass of cold water should never be taken without removing it from the lips. It has killed many a man. Grasp the whole glass in the palm of the hand, take one swallow at a time, remove the glass, then take another; in this way half a glass will satisfy quite as much as a whole one, and is perfectly safe, however warm the person may be.

